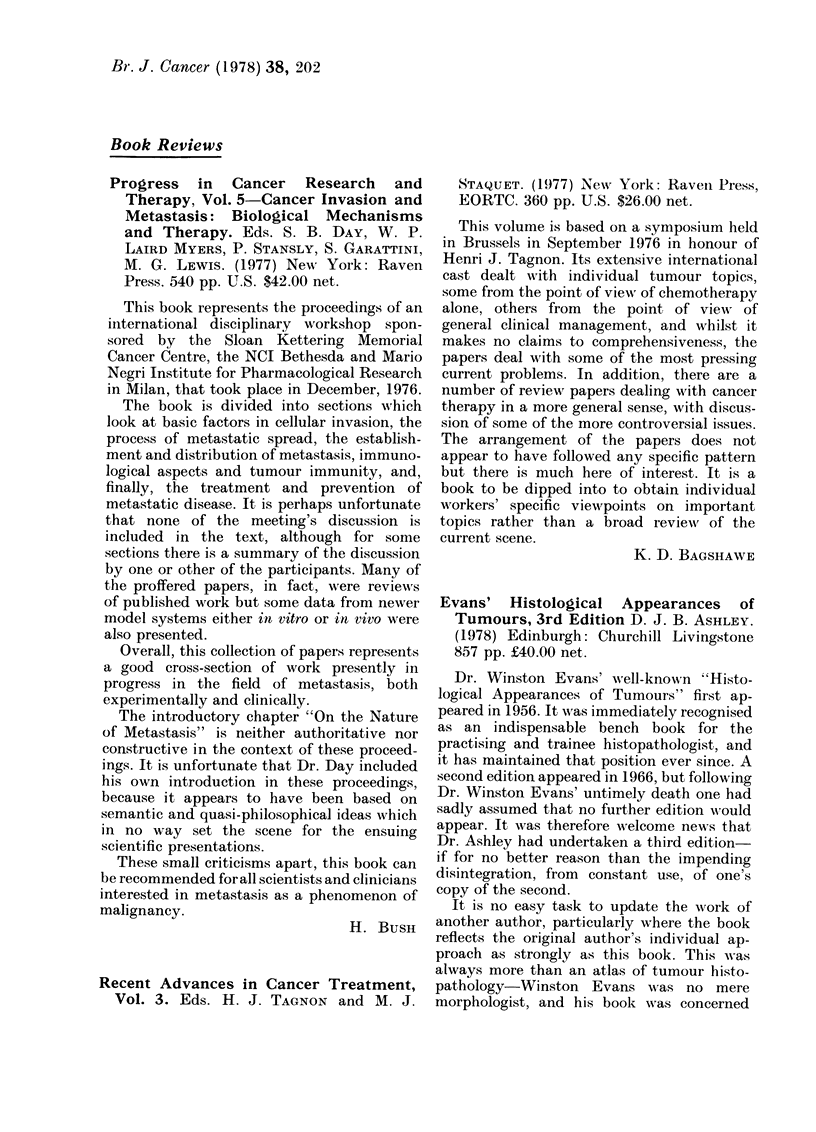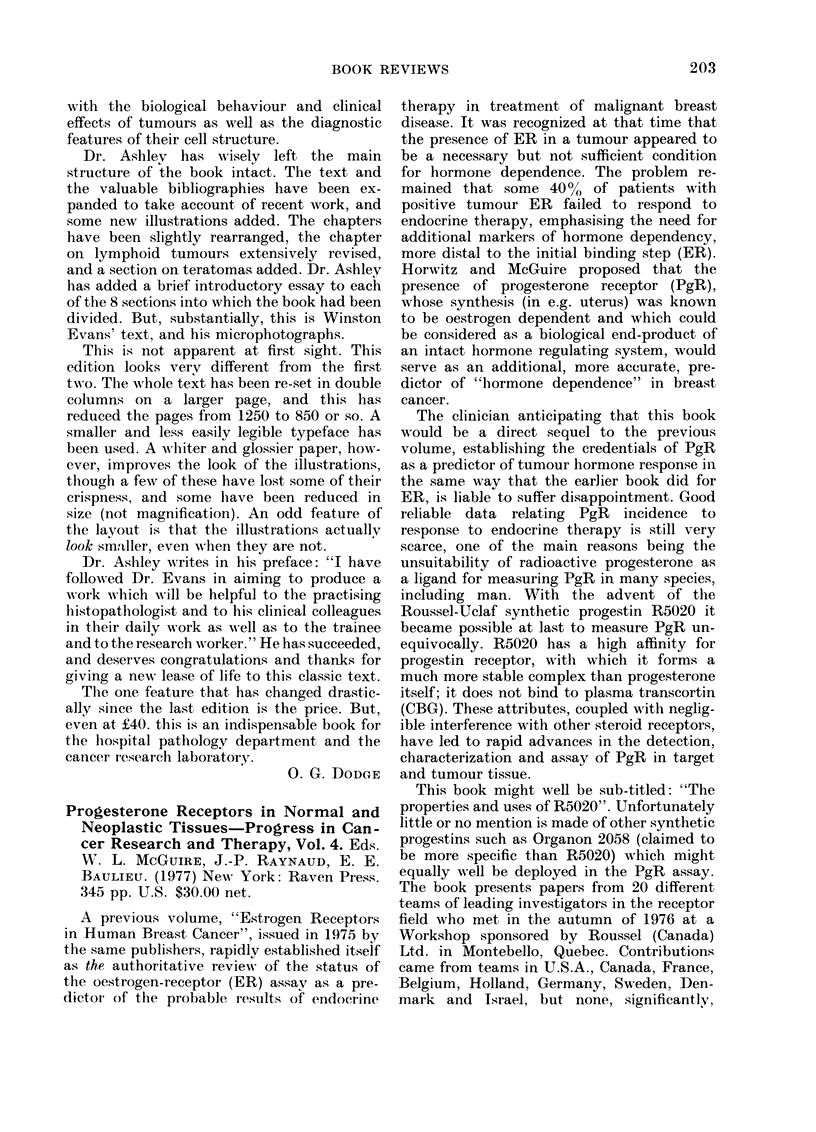# Evans' Histological Appearances of Tumours, 3rd Edition

**Published:** 1978-07

**Authors:** O. G. Dodge


**Evans' Histological Appearances of Tumours, 3rd Edition** D. J. B. Ashley. (1978) Edinburgh: Churchill Livingstone 857 pp. £40.00 net.

Dr. Winston Evans' well-known "Histological Appearances of Tumours" first appeared in 1956. It was immediately recognised as an indispensable bench book for the practising and trainee histopathologist, and it has maintained that position ever since. A second edition appeared in 1966, but following Dr. Winston Evans' untimely death one had sadly assumed that no further edition would appear. It was therefore welcome news that Dr. Ashley had undertaken a third edition—if for no better reason than the impending disintegration, from constant use, of one's copy of the second.

It is no easy task to update the work of another author, particularly where the book reflects the original author's individual approach as strongly as this book. This was always more than an atlas of tumour histopathology—Winston Evans was no mere morphologist, and his book was concerned with the biological behaviour and clinical effects of tumours as well as the diagnostic features of their cell structure.

Dr. Ashley has wisely left the main structure of the book intact. The text and the valuable bibliographies have been expanded to take account of recent work, and some new illustrations added. The chapters have been slightly rearranged, the chapter on lymphoid tumours extensively revised, and a section on teratomas added. Dr. Ashley has added a brief introductory essay to each of the 8 sections into which the book had been divided. But, substantially, this is Winston Evans' text, and his microphotographs.

This is not apparent at first sight. This edition looks very different from the first two. The whole text has been re-set in double columns on a larger page, and this has reduced the pages from 1250 to 850 or so. A smaller and less easily legible typeface has been used. A whiter and glossier paper, however, improves the look of the illustrations, though a few of these have lost some of their crispness, and some have been reduced in size (not magnification). An odd feature of the layout is that the illustrations actually *look* smaller, even when they are not.

Dr. Ashley writes in his preface: "I have followed Dr. Evans in aiming to produce a work which will be helpful to the practising histopathologist and to his clinical colleagues in their daily work as well as to the trainee and to the research worker." He has succeeded, and deserves congratulations and thanks for giving a new lease of life to this classic text.

The one feature that has changed drastically since the last edition is the price. But, even at £40. this is an indispensable book for the hospital pathology department and the cancer research laboratory.

O. G. Dodge